# Cascade plasmapheresis (CP) as a preconditioning regime in ABO-incompatible live related donor liver transplants (ABOi-LDLT)

**DOI:** 10.1186/2047-1440-3-17

**Published:** 2014-09-12

**Authors:** Aseem Kumar Tiwari, Prashant Pandey, Geet Aggarwal, Ravi C Dara, Ganesh Rawat, Vimarsh Raina, Arvinder Singh Soin

**Affiliations:** 1 Department of Transfusion Medicine, Medanta-The Medicity, Sector-38, Gurgaon 122001, India; 2 Laboratory Services and Transfusion Medicine, Medanta-The Medicity, Sector-38, Gurgaon 122001, India; 3 Department of Liver Transplantation and Regenerative Medicine, Medanta-The Medicity, Sector-38, Gurgaon 122001, India

**Keywords:** Cascade plasmapheresis, Plasmapheresis, Transplant, Titer, ABO-incompatible transplant, ABO-compatible transplant, ABO-incompatible live related donor liver transplants (ABOi-LDLT)

## Abstract

**Background:**

ABO-incompatible live donor liver transplant (ABOi-LDLT) is being widely done to bridge the gap of demand and supply of organs. Different desensitization regimes are being used to reduce titer of blood group antibodies for successful transplant and accommodation of graft. The authors used cascade plasmapheresis (CP) to bring down titer of naturally occurring blood group antibody to 16 or lower.

**Material and methods:**

Four recipients of ABOi-LDLT were of blood groups O, O, B, and B while donors were of blood groups B, A, AB, and AB, respectively. Desensitization protocol included immunosuppressive drugs and plasmapheresis. CP consisted of separating patient’s plasma as the first step and passing it through pore size based filter column as the second step. The first step was performed using disposable kit (PL1, Fresenius Kabi, Germany) with minor modification on apheresis equipment COM.TEC (Fresenius Kabi, Germany). Pore size based filter column used was 2A column (Evaflux, Kawasumi Laboratories, Japan). Blood group antibody titer (immunoglobulin G (IgG)) was done by column agglutination technology (Ortho-Clinical Diagnostics).

**Results:**

Cases 1, 2, 3, and 4 with pre-CP titer of 1,024, 512, 32, and 64 required four, three, one, and one CP procedures, respectively. No signs of antibody-mediated rejection were exhibited on histopathological evaluation by any of the patients. Successful organ engraftment occurred as documented by post-operative liver function tests and liver biopsy.

**Conclusion:**

Cascade plasmapheresis offers a cost-effective and efficient way to decrease blood group antibody titer and helps in successful transplant.

## Background

A large number of liver transplants are being performed in India, and majority is live donor liver transplant (LDLT) [1]. In India, the organ transplants are governed by Organ Donation Act [2], which allows only first-degree relatives or spouse to be donor(s) for the patient. Sometimes this willing donor is not suitable on the grounds of ABO blood group incompatibility. However, in recent times, people have found their way around this ‘suitability issue’ by doing ABO-incompatible (ABOi) solid organ transplants successfully using various desensitization protocols [3,4].

Desensitizing protocols play an important role in successful outcome of these transplants by decreasing the chances of acute antibody-mediated organ rejection [4]. These protocols include immunosuppressive drugs and plasmapheresis. Immunosuppressive drugs inhibit the formation of new antibodies, and plasmapheresis lowers the titer of existing blood group antibodies. There is a report from India reiterating pivotal role of plasmapheresis in desensitization protocols leading to successful solid organ transplant and adequate patient follow-up after the transplant, as well [5]. However, this was conventional plasmapheresis with removal of patient’s plasma, and volume replacement was done with normal saline and donor fresh frozen plasma (FFP) units. We would like to present a case series of four consecutive ABOi-LDLT patients where we used cascade plasmapheresis (CP) successfully as a part of preconditioning regime to reduce the titer of naturally occurring antibody in ABO incompatible LDLT.

## Materials and methods

### Patient and donor selection

Four patients with end-stage liver disease (ESLD) who did not have ABO-compatible donor in the family were enrolled for ABOi transplant program. They were explained about the process of ABOi-LDLT including the plasmapheresis protocol (CP) with its potential benefit in reducing antibody titer and possible adverse effects like citrate effect and changes in blood pressure, etc. Informed consent was obtained for CP from the patients. All the prospective donors underwent extensive medical and psychological assessment. The donors who qualified these assessments were briefed about the surgery, its duration, risks, length of stay in the hospital, etc. The donors then provided written consents for organ donation. The demographic profile, primary diagnosis, comorbid conditions, model for end-stage liver disease (MELD) score of these four patients, and demographic profile of their donors are given in Table 1.

**Table 1:** Profile of the patients and donors

**Patient**						**Donor**		**Titer**
Case number	Age and gender	Diagnosis	Comorbid conditions (indication for transplant	MELD score	Blood group	Relationship with patient	Blood group	Baseline titer
Case 1	33/F	HCV-related chronic liver disease (CLD)	Jaundice, ascites, PH	19	O positive	Sibling	B positive	2,048
Case 2	42/M	Crypotogenic CLD	Jaundice, ascites, HRS	19	O positive	Wife	A positive	1,024
Case 3	56/M	HCV-related CLD	SBP, HE, Ascites	18	B negative	Daughter	AB positive	64
Case 4	59/F	HCV-related CLD	Cholecystitis, PH , HCC	19	B positive	Daughter	AB positive	64

*PH* portal hypertension, *HRS* hepatorenal syndrome, *SBP* subacute bacterial peritonitis, *HE* hepatic encephalopathy, *HCC* hepatocellular carcinoma.

### Desensitization protocol

Desensitization protocol included immunosuppressant drugs and cascade plasmapheresis.

### Immunosuppressant drug regime

The drug regime was started with anti-CD20 drug (rituximab) which was administered as a single dose of 100 mg, 19 days prior to planned date of surgery to inhibit formation of new antibodies. Thereafter, plasmapheresis was initiated to remove the existing blood group antibodies till the titer of 16 or lower was achieved. The other three immunosuppressive drugs (mycophenolate mofetil, tacrolimus, and glucocorticoids) were initiated prior to the surgery as per the standard hospital protocol. Oral mycophenolate mofetil (MMF) 500 mg/twice a day was started 7 days prior to LDLT. Tacrolimus was initiated on the day of LDLT, and tacrolimus trough level was maintained between 10–15 ng/ml in the first 2 weeks, 7–10 ng/ml between 2–12 weeks, and 5–7 ng/ml until 6 months. Prednisolone was administered on the day of surgery at a dose of 2 mg/kg for 1 week, then at 1 mg/kg for 2 weeks, then gradually tapered to 0.5 mg/kg by fourth week, and stopped 3 months after surgery. MMF and tacrolimus were continued for life. These three drugs were also used in ABO-compatible (ABOc) liver transplants.

### Cascade plasmapheresis

**Figure 1:**
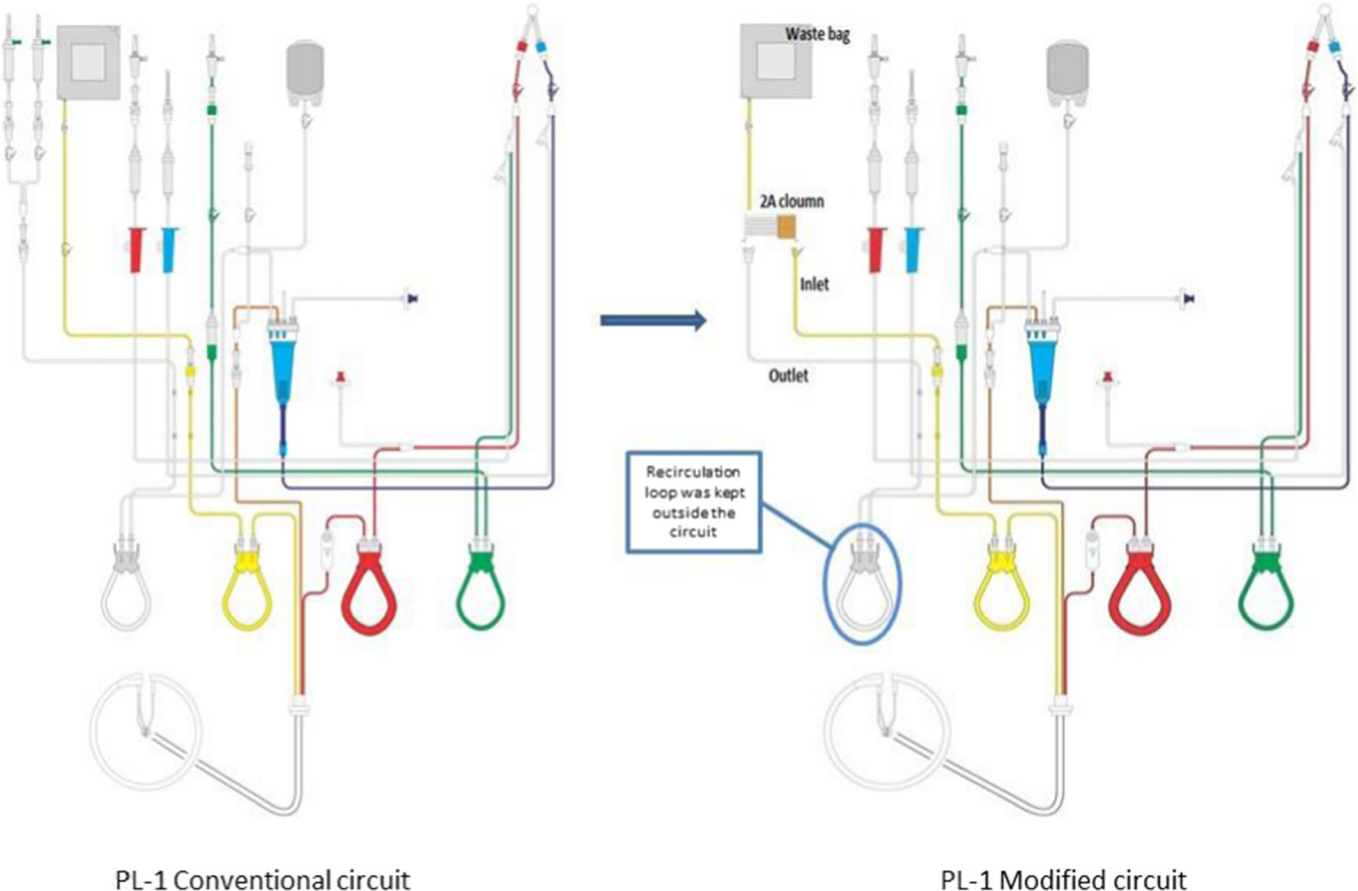
Conventional and modified PL1 circuits.

CP was initiated after an average of 19 days (range 11–24 days) after rituximab administration. CP consisted of separating patient’s plasma as the first step and passing it through a pore size based filter column as the second step. The first step was performed using plastic disposable kit (PL1, Fresenius Kabi, Germany) on the apheresis equipment COM.TEC (Fresenius Kabi, Germany). The pore size based filter column used was 2A column (Evaflux, Kawasumi Laboratories, Japan) [6]. This column comes with tubing which is compatible and was used in conjunction with PL1 kit. The attachments were made under sterile conditions (laminar airflow) in a manner that the separated plasma would be the ‘inflow’ to the filter and the filtered plasma would be ‘reinfusion’ in the PL1 kit and thus, completing the vein to vein circuit. Though the disposable kit prescribed by the manufacturer for such procedures is P1R, the authors adapted routine kit used for plasma exchange PL1 for CP procedures. In PL1 kit used for conventional plasmapheresis, the separated plasma is replaced via the replacement of fluid pump (white) generally at the ratio of 1:1. The loop of the kit which passes through the replacement fluid pump was kept outside the pump in the modified circuit (Figure 1). This minor modification was done since there was no need of 1:1 replacement. The replacement, if any, was done with 5% albumin through a separate intravenous line.

### 2A column

The pore size based column was a semi-selective column and manufactured in different pore sizes (2A, 3A, 4A, 5A). The 2A column performs well with separation of globulins and albumin. The sieving coefficient of the column for IgM is 0.0, immunoglobulin G (IgG) is 0.2, and albumin is 0.62. The sieving coefficient is defined as the ratio of a solute in the filtrate to the simultaneous concentration of the same solute in the plasma. In other words, sieving coefficient is the ratio of solute returned to the patient. The manufacturer markets the 2A column for various applications including nephrologic (rapidly progressive glomerulonephritis, Goodpasture’s syndrome, ABOi renal transplant), dermatologic (pemphigus vulgaris, bullous pemphigoid), systemic lupus erythematosus, neurologic (myasthenia gravis, Guillain-Barre syndrome, chronic inflammatory demyelinating polyradiculoneuropathy), etc. With the understanding of the principle, the authors used 2A column for decreasing the antibody titers in ABOi-LT.

### Titer

The blood group antibody titer was done by column agglutination technology (Ortho-Clinical Diagnostics). The cassettes used were anti-IgG, anti-C3d, and polyspecific (Ortho BioVue System, Ortho-Clinical Diagnostics, High Wycombe, UK), and the technique was low ionic salt solution-indirect antiglobulin test (LISS-IAT). The procedure was the same as published previously [5], and only IgG was considered to decide upon patient management. The titer was done before and after each CP procedure before surgery, daily for 7 days after surgery, and at least twice weekly till 6 weeks post-surgery.

### Ethical clearance

The protocols used for desensitization were ‘standard of care’. The study was purely an observational one, and therefore, no ethical approval was needed.

## Result

**Figure 2:**
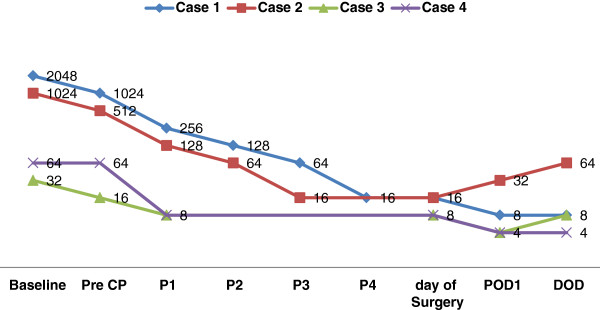
Changes in ABO antibody titers (Baselines titers = titer was done prior to rituximab administration; Pre-CP = Pre-cascade plasmapheresis, titer was done before the first CP; P = Procedure; POD = Post-operative day; DOD = day of discharge).

The desired titer of 16 or lower was achieved in one to four CP procedures. The cases 1, 2, 3, and 4 with the pre-CP titer of 2,048, 1,024, 32, and 64 required four, three, one, and one CP procedures, respectively. Figure 2 shows titer in the four patients, respectively. Titer of the three patients (except case 2) remained low (below 16) throughout the post-operative period. In case 2, the titer went up from 16 to 128 post-LDLT along with increment in liver enzymes necessitating intravenous immunoglobulins (IVIG) and three consecutive CP on post-operation day 2, 4, and 6.

Post-operative hospital course was largely uneventful. No histopathological evidence of antibody-mediated rejection was exhibited by any of the four patients in the biopsy performed at 1 month (protocol biopsy) post-LDLT. Successful organ engraftment occurred as documented by post-operative liver function tests, ultrasonography, and liver biopsy. Two out of four patients (case no. 3 and 4) had sinus ventricular tachycardia, two and 17 days post-LDLT, respectively, which was managed by intravenous diltiazem. Antibiotics administered prophylactically to all four patients were pipracillin-tazobactum and teicoplanin. Case no. 3 required additional antibiotics (linezolid, polymixin E, and meropenem) on post-operation day 3 based on culture and sensitivity of blood and endotracheal tube secretions. All the four patients were discharged after an average of 21 days (range 20–23 days) with dietary and medical advice.

Follow-up regime after discharge was once a week until 3 months, once a fortnight till sixth month, once a month till 2.5 years, and once every 2 months till life. Case 1 was readmitted on post-operation day 56 with acute pancreatitis. She succumbed despite 5 days of active medical management. All other three patients continue to do well with mean follow-up of 9 months.

## Discussion

Rituximab was given to all four patients to inhibit the formation of new antibodies and thus, decrease the titer of naturally occurring blood group antibodies. Plasmapheresis was subsequent to antibody titer decrease obtained by rituximab alone as evidenced by antibody titer monitoring. However, either there was no decrease in titers or at best decrease by one dilution. Plasmapheresis sessions achieved the desired levels of 16 or less thereafter. Plasmapheresis, therefore, remains an integral part of any desensitization regime.

Plasmapheresis can be conventional, cascade, or adsorption. While the conventional plasmapheresis is completely nonspecific with removal of entire plasma and its substitution with replacement fluid like FFP, albumin, normal saline alone, or more commonly in combination; cascade plasmapheresis is semi-selective with removal of high molecular weight substances mainly immunoglobulins; and some amount of albumin and adsorption column plasmapheresis is the most specific with removal of only ABO-specific immunoglobulins.

In India, solid organ transplant with conventional plasmapheresis is already reported [5]. Our series of ABOi solid organ transplants with CP with slight modification is probably one of the first such reports. The advantages of cascade plasmapheresis are that largely immunoglobulins are removed (100% IgM, 80% IgG) and most of the albumin and other plasma proteins (62%) are returned to the recipient. Though small amount of albumin is lost, it is much smaller as compared to conventional plasmapheresis. This also means that there is a very little need for replacement fluids. The mean amount of replacement fluid (5% albumin) required in our four patients was 500 ml (range = 250–700 ml). The lower volume of replacement fluid required in this series is in agreement with other published reports [7]. The procedure resulted in very little hemodynamic changes as exhibited by stable vitals of the patients monitored during the procedures. Further, as against the number of conventional plasmapheresis procedures required to achieve the desired titer, the number of CP procedures in our series was substantially fewer as shown in Table 2.

**Table 2:** Comparison between numbers of plasmapheresis procedure required as per guidelines and number of procedures in the present series and their costing

**Case (pre-CP titer IgG)**	**Number of conventional plasmapheresis as per guidelines**[8]	**Costs of conventional procedures in US dollars**	**Number of CP procedures in this series**	**Costs of CP in US dollars**	**Cost savings in US dollars**
Case 1 (2,048)	10–12	6,417	4	3,333	3,084
Case 2 (1,024)	10–12	6,417	3	2,500	3,917
Case 3 (32)	3	1,750	1	834	916
Case 4 (64)	4–5	2,625	1	834	1,791

**Figure 3:**
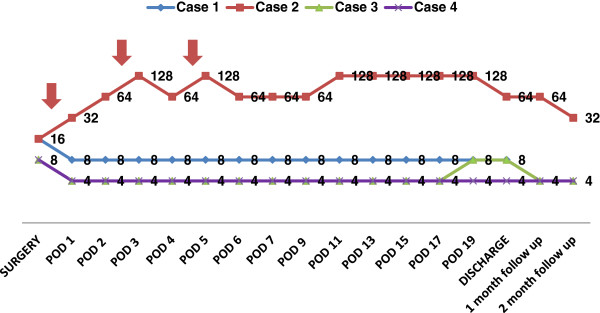
ABO antibody titers in post-surgical period.

This translated into multiple advantages for the patient. The patients could undergo early transplants which resulted in a shorter length of hospital stay, lesser chances of infection due to fewer interventional procedure, and better patient compliance. Though the disposable kit prescribed by the manufacturer for such procedures is P1R, the authors adapted routine kit used for plasma exchange PL1 for CP procedures. This was easily available since PL1 is used very often (for plasma exchange procedures), and it also meant that the authors did not have to maintain two separate inventories of P1R and PL1. Though individual CP procedure was more expensive as compared to conventional procedure ($834 vs. $584), there was cost saving since overall number of procedures in all four cases were substantially lower as compared to the possible number of conventional procedures (Table 2).Most centers would possibly not consider a patient with baseline titer higher than 512 for ABOi transplant. The authors had to take two patients for ABOi transplants with titer higher than 512 (2,048 and 1,024) since there were no suitable ABOc donors, and patients’ condition were deteriorating. Post-operatively titers were monitored daily, and in three patients, they remained low (<16). However, in one patient (case 2), the titers rebounded 2 days after surgery with increment in liver enzymes. This necessitated IVIG and three CP procedure on alternate days for 6 days before the patient stabilized and the liver enzymes came down to normal values. Though the titer in case 2 continued to remain high (Figure 3), the patient did well as per clinical and lab parameters.

Immediate post-operative follow-up of patients revealed ventricular tachycardia in two out of the four patients, which could be managed easily by antiarrhythmic agent. One patient had to be put on higher antibiotics based on culture reports to which the patient responded well. One patient (case 1) was readmitted because of acute pancreatitis and died after successful discharge. Larger series of patients would establish long-term safety of such patients.

## Conclusion

ABO blood group hurdle has now been crossed, and ABO-incompatible solid organ transplants are being successfully performed in India and other countries. ABO-incompatible liver transplant can and should be an option for seriously ill patients, who are awaiting transplantation and should be offered to all patients in cases of immediate need of allograft and are without any blood group compatible organ donor. Cascade plasmapheresis using modified PL1 offers a cost-effective and efficient way to decrease the blood group antibody titer and helps in successful transplant.

## Competing interests

The authors declare that they have no competing interests.

## Authors’ contributions

AKT, PP, GA, and RCD participated in the design, data acquisition, analysis, and interpretation. AKT and GA wrote the manuscript. GR performed most of the apheresis procedures and data compilation. VR and ASS were involved in coordination of the study and revision of the manuscript. All authors read and approved the final manuscript.
